# Incidence of post-partum complications and referrals of mothers and neonates to hospitals from a Midwife Obstetric Unit

**DOI:** 10.4314/ahs.v24i2.27

**Published:** 2024-06

**Authors:** Akm Monjurul Hoque, Somaya Buckus, Maariyah Hoque

**Affiliations:** 1 Kwadabeka Community Health Centre, South Africa 4 Khuleka Drive, Clernaville, 3061; 2 South African College of Applied Psychology, South Africa

**Keywords:** Antenatal care, gestational age, syphilis

## Abstract

**Background:**

The successful implementation of obstetric care should identify the maternal and foetal complications and refer to higher healthcare facilities in saving their lives. The study aimed to estimate the maternal and foetal complications risk factors during post-partum.

**Method:**

A retrospective cohort study was undertaken at a midwife obstetric unit among all women who had childbirths from January 2018 to October 2019. Regression analysis was used to predict risk factors.

**Results:**

The maternal and neonatal complications were 5.9% and 6.7% respectively. Regression analysis showed that mothers did not have antenatal care (ANC) were 2.8 times (OR=2.8, 95% CI: 1.5:5.4, p=0.001) and six times (OR=5.9, 95% CI; 2.7:12.5, p=0.000) more likely to have maternal and neonatal complications respectively. Gestational age < 32 weeks 19.0 times, (OR=19.0, 95% CI; 9.3:39.0, p=0.000) and 32-36 weeks, 4.6 times (OR=4.6, 95% CI; 2.5:9.4.0, p=0.000) more likely to have neonatal complications. Mothers without syphilis was 63% (OR=.37, 95% CI; .14:.97, p=0.04) less likely to have neonatal complications.

**Conclusion:**

Maternal and neonatal complication rates were comparable with others of similar settings. Pregnant women should be educated on the importance of ANC and strategies should be considered for improving ANC uptake and care to reduce maternal and neonatal complications.

## Introduction

Maternal and neonatal complications resulting from referral from a lower level e. g., Primary Health Care (PHC) to higher level healthcare facilities is a common practice in South Africa (SA) based on national guidelines.^1^ Maternal mortality ratio (MMR) and perinatal mortality rate are commonly used to measure maternal and perinatal health as highlighted in the United Nations Millennium development goals (MDGs) and recent initiative of Sustainable development goals (SDG).^2^ However, maternal death is considered the tip of the iceberg of maternal morbidity. It is estimated that for every maternal death, there are 20 to 30 incidence of maternal acute or chronic conditions or complications related to pregnancy and childbirth.^3^-^5^ Maternal morbidity working group (MMWG) of World Health Organization (WHO) defined maternal morbidity as any health condition attributed to and/or complicating pregnancy and childbirth that has a negative impact on woman's wellbeing and functioning.^6^ Referral is defined as the movement of healthcare users requiring a higher level of care from the existing level of care in the health service delivery system.^7^ The successful implementation of functional obstetric care that can identify the maternal or foetal complications and referral system are beneficial in saving the lives of both the mothers and neonates as pregnancy-related complications are unpredictable and can rapidly progress to morbidities and mortalities.^7^,^8^ A significant number of pregnant women are having childbirths at midwife obstetric units (MOU) in SA. Between 25% to 30% of all deliveries are taking place in MOU showed in the latest SA Demographic and health Survey (2019) and other reports,^9^,^10^ MOUs from Community Health Centres (CHC) and Primary Health Care (PHC) facilities are designed to deliver low-risk pregnant women.^1^

Maternal deaths resulting from pregnancy complications are found to increase from 7.2 to 18.0 per 100 000 live births from 1987 to 2014.^11^ In 2017, the MMR for SA was found to be 119 per 100 000 live births, and in 2019, the infant mortality rate of 25 per 1000 live births.^12^,^13^ The latest SA saving mother's report highlights that identification of maternal complications during labour and post-partum period and referral from MOUs and district hospitals are major contributors to institutional maternal deaths 43% and 50% respectively and while 36% of all maternal deaths occur in the postpartum period.^14^ According to a report from Tunisia, majority (85%) of maternal deaths are considered or classified as preventable as half of them are attributed to the failure to identify complications and delay in referring to higher healthcare facilities.^7^ Similarly, it is found from SA that significant numbers of maternal and neonatal mortalities are preventable if efficient, effective and timely identification of complications and referrals to appropriate level of healthcare facilities by health care workers are undertaken.^15^

The maternal and neonatal mortality rates in SA has not reached the targets of MDG 4 (to reduce child mortality) and MDG 5 (to improve maternal health).^16^ The risk factors for higher maternal mortality in SA are known as non-pregnancy related infection, hypertensive disorders of pregnancy such as pregnancy induced hypertension, pre-eclampsia, eclampsia, antepartum and postpartum haemorrhage..^17^ The major contributing factors for neonatal and infant mortality are birth asphyxia, preterm births and congenital abnormalities but intra-partum asphyxia alone is responsible for 31% and 18% of babies born with birth weight < 2.5kg and > 2.5 kg babies respectively.^10^,^17^ It is also reported that the delay in identifying and referring babies for with complications to secondary or tertiary hospital was related to 8% of all stillbirths in SA.^10^ In response to the avoidable maternal and neonatal mortality, the SA National Department of Health (NDOH) has developed and implemented the Maternal, Perinatal and Neonatal (MPN) Health Policy that aims to reduce MPN morbidity and mortality rates by 50% by 2030, in accordance with the SDGs.^2^,^18^ The attendants of the pregnant mothers at the primary healthcare facilities need to timeously identify and respond to complications and obstetric emergencies by facilitating the appropriate referral to higher level of expertise care to improve the outcomes of mothers and neonates. The maternal care at a MOU start with a midwife as the initial contact who should be able to manage normal childbirths and able to identify complications and emergencies that require referrals.^15^

Little is known about pregnant women delivering at a MOU, the medical or obstetric and foetal complications during and after childbirth requiring referrals considering that timely identification of such conditions are fundamental in reducing maternal and neonatal morbidity and mortality. The MMWG of WHO has recommended that maternal morbidity and complications are measured at the PHC level from low-and-middle income (LMIC) countries for local level decision making for appropriate interventions.^5^ The objectives are to estimate post-partum complications of mothers and neonates, risk factors and referrals to higher healthcare facilities.

## Methods

### Study design

A retrospective cohort study was accomplished aiming at all women who have had live child births, from January 2018 to October 2019.

### Study setting, population and sampling

The study was undertaken at a MOU of a PHC facility. The facility is situated in a peri-urban community, about 40Kms of the outer west region of Durban metropolitan city in SA. It serves predominantly black residence of over 150 000. Most of the residents are poor, living in mainly informal types of dwellings. The facility provides a full package of PHC services including a full range of reproductive health, including maternity services. Maternity services include antenatal care, delivery and post-delivery services that are based on national guidelines.^1^ High-risk pregnancies defined by the SA national guidelines are referred to hospitals for further antenatal care (ANC) and delivery. These hospitals are St. Mary's (SMH) for district level care for mothers and neonates, Addington (ADH) and King Edward viii (KEH) hospitals for maternal regional and tertiary level care and R K Khan Hospital for neonatal regional level care. Maternity services at the MOU are available 24 hours a day, free of charge and run by midwives and supported by a residence medical officer. According to the national guidelines, the MOU is responsible for antenatal care for low-and intermediate- risk pregnancies, treatment of common problems of pregnancy-related conditions, management of labour and delivery services, postnatal care and the management of emergencies at any stage of pregnancy and delivery. Only vaginal deliveries are conducted at the MOU without the use of any instrument (e. g., forceps, vacuum or caesarean section) and without any induction or augmentation of labour. The study population for this study were all women who had childbirths and all live birth babies for the study period.

### Labour ward practices

When a pregnant woman attends the labour ward with labour pain, a proper history is taken, examinations and investigations are conducted to diagnose or classify any obstetric and foetal problems and the risks of pregnancy and delivery. Labour is defined with painful uterine contractions accompanied by effacement and dilatation of the cervix and or with the presence of show and or rupture of the membrane. Women with active labour without any apparent or imminent complications are then admitted and allowed to continue to deliver at KCHC using a partogram (a chart with entering all maternal and foetal observations, fluids intake and output and medications) to monitor progress of labour. Alert and action lines on the partogram together with other observations (e.g., temperature of mother, BP, foetal heart rate etc.) are used to identify labour complications of mothers and the foetuses during labour. In cases of complications or risk factors (e.g., raised BP of mothers, eclampsia, foetal distress, etc.) identified based on national guidelines during different stages of labour or delivery. In case of maternal or foetal complication that can't be managed at the MOU a telephonic presentation and discussion with the medical practitioner to the referring hospital is carried out by the midwife. The mother is then transported to the hospital using Emergency Medical Rescue Service's ambulance. For those that deliver at, observations are done for 8 hours after delivery. Any complications to mothers and neonates are also referred to hospitals. Mothers and the neonates without any complications are discharged home with proper counselling for neonatal care, vaccinations, breastfeeding, family planning etc. The complications during post-partum period are listed in the guidelines for hospital referrals and those are: retained placenta, postpartum haemorrhage (PPH), delivery of incomplete placenta or suspected retained products of conception, inverted uterus, anaemia (Hb <8 g/dL), maternal pyrexia ≥ 37.5 degrees Celsius, third- and fourth- degree perineal tears and hypertension disorders.^1^

### Definition of terms

The maternal and neonatal complications are defined as those conditions that adversely affect mothers and their neonates during and after delivery. Those conditions are managed adequately without any substantial effort on the woman and the neonate. Neonatal health are not classified in this study. For example, first- and second-degree perineal injuries and episiotomies are some of them. However, the third-and fourth degrees perineal injuries require expertise to repair and thus considered as a complication that occurred during childbirth.

Postpartum haemorrhage (PPH) is considered when the mother had excessive vaginal bleeding within 8 hours after delivery and the mother becomes hypothermic (<35°C) and or the mother became unconscious.

Retained placenta is considered when the placenta did not detach and or come out for more than one hour after the childbirth.

Maternal anaemia is considered when the mother's (ward) haemoglobin was < 8 grams/dl measured with a haemoglobinometer.

Hypertensive disorder is grouped (defined) when the mother's blood pressure remained persistently ≥150/100 mmHg or symptomatic for imminent eclampsia or eclampsia after delivery.

APGAR stands for Activity, Pulse (heart rate), Grimace (response to stimulation), Appearance (colour) and Respiration (breathing). Each category is scored with 0, 1 and 2, depending on the observed condition. The highest possible score is 10. It is a quick test or observation performed on a baby at 1 and 5 minutes after birth. The APGAR scores in one-minute determines how well the baby tolerated the birthing process. The scores in 5-minutes indicate the healthcare provider actions on the baby outside the mother's womb. A baby with a lower score after 5 minutes has a higher risk for neonatal asphyxia. Interpretation of APGAR scores are: scores between 7 to 10 after five minutes are “reassuring” and the scores between 4 to 6 “moderately abnormal” for neonates require hospital referrals. If the APGAR score of a baby is low at one minute, an intervention is warranted.

Birth asphyxia is defined when the neonate's APGAR score remains <8 after 5 minutes after delivery.

Preterm or prematurity of babies are defined with babies born of mothers <37 weeks GA. Babies born premature without any complication such as good APGAR and or without birth asphyxia were not considered for referrals in this study.

Birth weight < 2.5 kilograms is considered as low-birth-weight deliveries (LBW). LBW babies with low APGAR score with or without respiratory distress were considered complications for referrals.

### Data collection

Data was collected from the birth register by the research assistants. The birth register was developed and implemented by the NDOH, and it was the only official register for all births and deliveries, referrals (if required after delivery) and discharges. The register contained among other variables the name, age, parity, number of antenatal visits, time of admission, delivery and discharge of the mother and maternal deaths, and pregnancy outcomes (live births, fresh/macerated still-births, neonatal deaths), APGAR scores in 1 and 5 minutes, birth weight of the baby etc.. The delivering midwives entered these data (variables) in the birth register.

### Data analysis

Data was collected in Microsoft excel, transported, coded and analysed using SPSS 22.0 for window version. The baseline demographics and outcome variables were summarized using descriptive summary measures: expressed as mean with standard deviation (SD) for continuous variables and percent for categorical variables. The outcome variables for maternal and neonatal complications were presented with proportions of complications and percentages of deliveries and live births respectively. Cross table analysis of outcome variables (maternal and foetal complications) with maternal independent variables using Pearson's Chi-square (X 2) test and p values were undertaken to identify significant variables with the outcome variables. Variables found significant (p<0.05) were used in step by step (backward) logistic regression analysis to identify significant predictors for the outcome variables. All statistical tests were performed using two-sided tests at the 0.05 level of significance. For regression models, the results were expressed as an effect with adjusted odds ratios (OR) for binary outcomes with corresponding 95% confidence intervals (95% CI), and associated P values. P value <0.05 was considered significant.

### Ethical considerations

We obtained ethical permission from UMgungundlovu Health Ethics Review Board (Reference no. UHERB 015/2020). KZN Health Research Committee and the management of the health facility gave permission to use the relevant data for the study. Informed consent was waived by the ethics committee as it was secondary data.

## Results

A total of 1486 pregnant women delivered 1460 live birth babies (and 26 were still births) during the study period (3 of them had twin pregnancies) out if which 57 (3.8%) women delivered at home or on the way to the facility (baby born before arrival (BBA) are included in the study). Demographic and pregnancy related information including outcome variables is presented in Table 1. The teenage pregnancy rate was 14.8%. More than half (58.6%) of the mothers were between 20 to 29 years of ages. Almost one third of them (30.7%) had parity 1, and over half of them had parity between 2 and 3. Only a few (1.3%) had higher parity (> 5). Majority (93.2%) of these women received ANC during pregnancy. The mean ANC visit was 5.46 (SD=3.02) and majority (68.8%) of them received 4 or more ANC visits. Most of the pregnant women (84.6%) delivered at term (> 37 weeks of gestation) while 15.4% were preterm deliveries. The prevalence of HIV and syphilis at birth were 44.2% and 2.4% respectively among these women.

**Table 1:** Frequency distribution of demographic, antenatal and cause of referrals

Variables	Frequency	Percentage
**Age (n=1485)**		
< 19 years (Teenage pregnancy)	220	14.8
20 - 24 years	427	28.8
25- 29 years old	436	29.4
30- 34 years old	280	18.9
=> 35 years old	122	8.2
**Mean age (SD)**	**25.9 (5.70)**	
**Parity (n=1480)**		
Nil parity	455	30.7
1-2 parity	818	55.3
3-4 parity	188	12.7
=>5 parity	19	1.3
**Booked before 20 weeks of Gestation (n=1460)**		
No	691	47.3
Yes	769	52.7
**Number of antenatal visits (n=1447)**		
Un booked (0)/No antenatal care	99	6.8
1-3 visits	260	18.0
4-7 visits	732	50.6
=>8 visits	356	24.6
**BBA (n=1486)**	57	3.8
**Gestational age at birth (n=1416)**		
< 32 weeks (preterm)	54	3.8
33-36 weeks	164	11.6
> 37 weeks	1198	84.6
**Birth defects (n= 1412)**	**15**	**1.1**
**HIV status (n=1480)**		
Positive	654	44.2
Negative	826	55.8
**Syphilis status (n=1481)**		
Positive	35	2.4
Negative	**1446**	**97.6**
**APGAR in 1 minute (n=1397)**		
Poor(<7)	65	4.7
**APGAR in 5 minutes (n=1399)**		
Poor (<7)	47	3.4
Maternal complications (n=1486)	88	5.9
Neonatal complications (n=1460)	98	6.7
**Maternal referrals to hospital types (n=88)**		
District hospital	30	34.0
Regional hospital	58	66.0
**Neonatal referrals to hospital types (n=98)**		
District Hospital	66	67.3
Regional hospital	32	32.7

A total of 88 mothers had developed complications after delivery that estimated the incidence of maternal complication 5.9% of all deliveries. The most common (Table 2) complication was maternal anaemia (26.2% of the complications) and the incidence of 1.5% of all deliveries, followed by PPH (20.5% of complications) and incidence of 1.2% all deliveries. The perineal injuries and hypertensive disorders were equally 17.0% of all complications with the incidence of 1.0% of all deliveries. Perineal injuries required hospital referrals for those had third- and or fourth-degree perineal injuries known as obstetric anal sphincter injury (OASI). The rate of OASI thus became 1% of all vaginal deliveries. Other maternal complications were 19.3% and the incidence of 1.1%. The other conditions were mainly retained placenta, retained products of conception, etc. Some women had more than one complication but they were counted only once, regardless the number of complications they may have had. Most of the mothers (66%) that had complications were referred to regional hospitals (ADH and KEH).

**Table 2:** Distribution of maternal and neonatal complications

Variables	Frequency	Proportion of complications	Incidence per 100 NVDs
**Maternal complications (n=1486)**	88	**100**	5.9
Maternal anaemia	23	26.2	1.5
Perineal injury	15	17.0	1.0
Hypertensive disorder	15	17.0	1.0
Other maternal cause	17	19.3	1.1
**Neonatal complications (n=1460)**	**98**	**100**	**6.7 per 100 live births**
Premature baby	42	42.9	2.1
LBW	16	16.3	1.1
Birth Asphyxia	9	9.2	0.6
Congenital abnormality/birth defects	17	17.3	1.2
Other neonatal conditions	14	14.3	0.9

A total of 98 neonates (incidence of 6.7% of all live births) had complications. Prematurity was the commonest complication of neonates (42.9% of complications and incidence of 2.8% of live births) followed by congenital abnormalities of 17.3% (of the complications) and incidence of 1.1% of live births. The LBW babies were the third commonest complication (16.3% of complications) and incidence of 1.1%. Other neonatal complications were 14.3% (of neonatal complications) and the incidence of 0.9% of live births.). Most of the neonates had complication (67%) were referred to district hospital (SMH).

Table 3 showed that maternal ANC booking status (yes or no) and number of ANC visits had significantly different rates (p<0.05) of maternal complications and thus entered into logistic regression model to predict for maternal complications. Similarly, maternal ANC booking status, number of ANC visits, initiation of ANC before 20 weeks of GA (yes or no), GA at birth, maternal syphilis status, APGAR scores of neonates at one (1) and five (5) minutes had significantly (p<0.05) different neonatal complication rates and thus entered into logistic regression model.

**Table 3:** Background factors and characteristics of pregnant women in relation to maternal and neonatal complications using Pearson's χ^2^ test and p values

Variables	Maternal complications		Neonatal complications	
**Maternal age (n=1485)**	**Percent**	**P values**	**Percent**	**P values**
< 19 years (Teenage pregnancy)	13.8	0.426	17.5	0.364
20 - 24 years	31.0		25.8	
25- 29 years old	33.3		23.7	
30- 34 years old	11.5		20.6	
=> 35 years old	10.3		12.4	
**Parity (n=1480)**				
Nil parity	35.6	0.424	32.3	0.985
1-2 parity	55.2		54.2	
3-4 parity	9.2		12.5	
=>5 parity	0.0		1.0	
**Booked for Antenatal care (n=1447)**				
Not booked	14.9	0.001	19.6	0.000
Booked	85.1		80.4	
**Booked before 20 weeks of Gestation (n=1460)**				
No	51.2	0.469	61.3	0.005
Yes	48.8		38.7	
**Antenatal visits (n=1447)**				
0 (No visit)	15.9	0.011	20.9	0.000
1-3 visits	17.1		18.7	
4-7 visits	45.1		46.2	
=>8 visits	22.0		14.3	
**Gestational age at birth (n=1416)**				
< 32 weeks (preterm)	7.6	0.196	27.3	0.000
33-36 weeks	11.4		27.3	
> 37 weeks	81.0		45.5	
**HIV status (n=1480)**				
Positive	52.3	0.119	49.5	0.280
Negative	47.7		50.5	
**Syphilis status (n=1481)**				
Positive	10.3	0.000	6.2	0.023
Negative	89.7		93.8	
**APGAR in 1 minute (n=1397)**				
Poor (<7)	13.3	0.000	28.3	0.000
Good (7-10)	86.7		71.7	
**APGAR in 5 minutes (n=1399)**				
Poor (<7)	10.8	0.000	15.2	0.000
Good (7-10)	89.2		84.8	

We found in the logistic regression analysis (Table 4) that those mothers did not book for antenatal care were 2.8 times (OR=2.8, 95% CI: 1.5:5.4, p=0.001) more likely to have maternal complications during the post-partum period. Logistic regression output for neonatal complications showed (Table 5) that the number of mothers' antenatal visit was a significant predictor for neonatal complications. Pregnant woman that had no antenatal visit was almost six times (OR=5.9, 95% CI; 2.7:12.5, p=0.000) more likely to have an overall neonatal complications compared to those that had 8 or more antenatal visits. GA was also a significant predictor for neonatal complications. GA < 32 weeks where 19.0 times, (OR=19.0, 95% CI; 9.3:39.0, p=0.000) and GA 32-36 weeks 4.6 times (OR=4.6, 95% CI; 2.5:9.4.0, p=0.000) more likely to have neonatal complications. Maternal negative syphilis status were 63% (OR=.37, 95% CI; .14:.97, p=0.04) less likely to have neonatal complications. Low APGAR scores (<7) in 1 minute were 5.4 times (OR=5.4, 95% CI; 2.51:11.94, p<0.001) more likely to cause foetal complications. APGAR scores > 7 in 5 minutes reduces the chance of neonatal complications by 90% the OR=.10 (95% CI; .023:.47, p=0.003).

**Table 4:** Logistic regression output for maternal complications

Variables	Significance (p value).	Adjusted odds ratio (OR)	95% CI for OR	
			Lower	Upper
Never Booked for ANC	.001	2.886	1.532	5.435
Constant	.000	.054		

Reference group: Booked for ANC

**Table 5:** Logistic regression output for neonatal complications

Variables	Significance (p value).	Adjusted odds ratio (OR)	95% CI for OR	
			Lower	Upper
No. of antenatal visits coded*	.000			
Antenatal visits 0	.000	5.904	2.783	12.522
Antenatal visits 1-3	.153	1.731	.816	3.669
Antenatal visits 4-7	.145	1.605	.850	3.032
GA coded*	.000			
GA < 32 weeks	.000	19.078	9.310	39.095
GA 33-36 weeks	.000	4.630	2.543	8.431
Mothers had no syphilis*	.044	.378	.147	.974
APGAR 1 min < 7*	.000	5.4	2.51	11.94
APGAR in 5 min > 7*	.003	.106	.023	.478
Constant	.000	.099		

* Reference group: Antenatal visits > 8, GA > 37 weeks, maternal syphilis positive, APGAR score > 7 in 1 minute, APGAR score < 7 in 5 minutes.

## Discussion

This study estimated the incidence and risk factors for maternal and neonatal complications at a MOU that required hospital referrals to different levels (District and regional level hospitals) of healthcare at post-partum. The rates of postpartum maternal and neonatal complications were 5.9% and 6.7% respectively. Furthermore, we found higher prevalence of maternal HIV (44.2%) and syphilis (2.2%) infections and preterm deliveries (15.4%). A similar maternal complication rate at post-partum period of 5% was reported from rural Tanzania and a rate of 6.1% from a MOU in SA.^19^, ^20^ The preterm delivery rates of our study are similar to the rates reported from a birth cohort study of poor peri-urban communities of SA where they found 17% preterm deliveries and 7% of all live birth babies had complications and required immediate hospitalisation after livebirths from two MOUs.^21^ The neonatal complications and preterm delivery rates in our study are also similar to the rates of 6.5% and 17.7% respectively found from Mofolo CHC in SA.^20^ However, the preterm delivery rate (15.4%) in our study is higher than the rate estimated (12%) for sub–Saharan Africa.^22^ The most common maternal complication requiring referral is maternal anaemia (26.2%) after delivery and it is similar to the rate (24.3%) reported from Ethiopia.^23^ PPH and perineal injuries accounted for 1.2% and 1.0% respectively in our study are similar to other report where 1.3% and 2.2% had PPH and perineal injuries from a CHC.^20^ However, the perineal injuries (OASI) in our study that required hospital referral is higher than the rate reported from Cape town (0.6%), but lower than the rate found in Durban hospitals of 4.1% in SA.^24^, ^25^ Complicated obstetric cases are delivered in hospitals therefore higher rate of OASI is expected in hospital deliveries than the rates found from MOUs delivering low-risk pregnancies.

The findings of the maternal complications conform to the findings of several other studies where maternal anaemia and PPH are the leading causes of maternal complications in the postpartum period and thus require the services of a higher level of health care.^23^,^26^-^28^ Maternal infections and sepsis, anaemia and PPH are the major causes of maternal deaths prompting the immediate management of such conditions as a priority and referral to well-equipped hospitals.^26^,^28^,^29^ Anaemia can have a significant impact in preventing maternal deaths that are due to PPH. It is shown that maternal deaths resulting from PPH are mainly due to undiagnosed and untreated anaemia and more prevalent in developing countries.^30^ PPH resulting in maternal mortality are 45% less likely to occur in hospitals with an obstetrician available than the MOUs.^18^, ^27^ This signifies that midwives should have a higher index of suspicion for maternal anaemia, which will prevent complications of anaemia (e. g. PPH) at post-partum period and to further reduce the need for referrals to higher healthcare institutions.

We found that women who had no antenatal care are 2.8 and 5.9 times more likely to experience maternal and neonatal complications respectively. Similar findings are reported in a study where pregnant women with insufficient ANC visits were 2.4 and 2.2 times more likely to experience severe maternal and neonatal complications respectively.31 Women not having ANC prior to delivery had double (OR = 1.8) the chance of having complications during labour and delivery compared to those who had 4 or more ANC visits.^31^ Pregnant women from sub-Saharan African countries are found with poor knowledge on the importance of antenatal visits and are at higher risk of both maternal and neonatal complications.^32^ A report from Ethiopia found that pregnant women who had four or less ANC visits are 2.4 and 4.4 times more likely to experience anaemia and postpartum haemorrhage respectively in the postpartum period compared to those had > 4 ANC visits.^23^ Benefits of antenatal care attendance have been found with statistically significant association of markedly positive pregnancy outcomes opposed to those that did not.^33^ Pregnant women in the above-mentioned study found that they where more prone to have negative foetal outcomes with no or fewer ANC visits such as low-birth weight infants, foetal deaths and preterm births. A study conducted in Pakistan concur that women who utilize ANC services are more than four times likely to give birth to infants of normal birth weight.^34^ Our finding of maternal complication and referral rate is concurrent with findings in a study conducted in the most populous state of India. 35

Obstetric complications related to hypertensive disorders (post-partum hypertension, pre-eclampsia and eclampsia) were not common in our cohort. The low incidence (1% of all deliveries and 17% of all maternal complications) of hypertensive disorders at immediate post-partum period (within 8 hours) may reflect that mothers were younger with a median age of 26 years and is similar to other findings from SA.^20^, ^36^

Mothers with complications are at risk of developing neonatal complications and deaths, especially in those mothers that deliver prematurely. 36 In this study, the common neonatal complication requiring referral is prematurity (22.5%), where mothers had delivered before 37 weeks of GA. Born in early GA (< 32 weeks) is found to be a statistically significant predictor (OR=19.0, p<0.05) of neonatal complications in our study. Early gestational age (<32 weeks) is known to be associated with LBW, severe intrauterine growth restriction and severe foetal hypoxia are more likely to be born prematurely with a greater risk of neonatal complications.^37^ As the GA increases, the growth of the foetus proportionately becomes mature and gain weight in normal circumstances. Delivery of neonates at lower GA led to prematurity and low birth weight babies. The highest proportion of babies (22%) was found premature and required referred to hospital as they needed special equipment and specialist care. Thus, the higher odd ratios for lower gestational age (OR=5.4) for GA <32 weeks and (OR=2.5) for 33-36 weeks respectively are justified and similar to other studies.18 Birth asphyxia is known to cause many neonatal deaths during the first day of life. 38 Saving Babies Report from SA has highlighted that baby's birth weight between 1.0 kg to 2.5 kg category, 18 per cent die due to intra-partum asphyxia, which is an indicator of poor intra-partum care. This is also the main cause of deaths for babies >2500g (30.8 per cent of all deaths) in SA. It is found that among neonatal deaths of term babies (>2.5 Kg,) birth asphyxia was the leading cause (64.9%) of neonatal deaths in SA. 14 It has been mentioned in a report that the infant mortality is increased in LBW infants subsequent to premature deliveries born in first level healthcare institutions and thus concluded that as the level of a healthcare facility decreases, the rate of infant mortality increases and vice versa.^38^ The LBW rate is also an indicator of the social determinants and socio-economic status of a region or community. The LBW delivery rates reported from African countries are higher of 13.8-17.4 and for SA 17.4 per 100 deliveries16. Not all LBW babies from this study are found with complications and referred to hospitals. Further to that LBW babies are classified with prematurity and complications were referred to hospitals. Pregnant women with low gestational age who presented with labour were referred to hospitals before delivery since they were likely to result in LBW.^1^

Our study further indicates that statistically significant predictors for foetal complications are mothers not attending ANC (OR=5.9), low APGAR scores (<7) at 1 minute (OR=5.4) and at 5 minutes. Good APGAR score (>7) at 5 minutes is found with 90% (OR=.10) resulting in reduction of neonatal complications. This is an indications of midwife's effort or intervention to act quickly to improve the condition of the neonate and thus improved APGAR scores and reduced neonatal complications and referrals.

HIV status in our study was not a risk factor for maternal and or foetal complications at post-partum period. It could be due to universal access to ART for pregnant women. However, ART was a known risk factor for maternal anaemia.^39^ Maternal syphilis status at birth was a significant predictor for foetal complications and are similar to our findings. It is also known that women and infants that are referred for higher level of care are at higher risk of poorer outcomes.^40^ The implementation of an efficient training programme for identification of maternal and neonatal complications and referral system are crucial in enhancing the quality of care and reducing maternal and neonatal morbidity and mortality.^38^ Midwives in the MOU must be able to timeously identify and promptly deal with the complications and refer mothers and or neonates at higher level of health facilities (hospitals) to minimize adverse outcomes.

## Strengths and limitations

Certain maternal physical and mental health factors during pregnancy may influence both maternal and neonatal health during puerperium (within 42 days) and later part of live. For example, in our analyses, HIV status did not significantly impact on any of main maternal and foetal complications, however, there are concerns about subsequent impacts of HIV and ART on maternal and child development. These impacts should be further examined in a longitudinal cohort study. Additionally, causality cannot be inferred due to our study design (retrospective cohort) thus we could not include comprehensive range of variables such as maternal sociodemographic, food insecurity and substance use in our study and analysis. Subjective bias is also possible in assessing and scoring APGAR. One midwife could score a neonate a “7” while another could score a “6”. This is why the Apgar score is just one of several assessments used to evaluate a newborn's general condition.

## Conclusion

The maternal and neonatal complications and hospital referral rates after delivery in our study are similar to the rates reported from other MOUs in SA. This report will improve staff morale of MOUs as they are identifying complications and referring to hospitals with similar rates. Indicators to monitor maternal and neonatal complications and referrals should be incorporated into plans for monitoring quality of obstetric care.
